# Preparation and characterization of spiked gold nanobipyramids and its antibacterial effect on methicillin-resistant *Staphylococcus aureus* and methicillin-sensitive *Staphylococcus aureus*

**DOI:** 10.1186/s43141-023-00589-4

**Published:** 2023-11-15

**Authors:** Phat Trong Huynh, Khanh Thi Le Tran, Tham Thi Hong Nguyen, Vinh Quang Lam, Ngan Thi Kim Phan, Thanh Vo Ke Ngo

**Affiliations:** 1 Research Laboratories of Saigon Hi-Tech Park, Ho Chi Minh City, 700000 Vietnam; 2 Faculty of Physics and Engineering Physics, University of Science, Vietnam National University Ho Chi Minh City, Ho Chi Minh City, 700000 Vietnam; 3 Vietnam National University Ho Chi Minh City, Ho Chi Minh City, 700000 Vietnam

**Keywords:** Spiked gold nanobipyramids, Gold nanobipyramids, Methicillin-resistant *Staphylococcus aureus*, Methicillin-sensitive *Staphylococcus aureus*

## Abstract

**Background:**

This paper reports the preparation of a new family of spiked gold nanoparticles, spiked gold nanobipyramids (SNBPs). This protocol includes the process to synthesize gold nanobipyramids (NBPs) using combined seed-mediated and microwave-assisted method and procedure to form spikes on whole surface of gold nanobipyramid. We also evaluated the antibacterial activity against both methicillin-resistant *Staphylococcus aureus* (MRSA) and methicillin-sensitive *Staphylococcus aureus* (MSSA) in various concentrations of SNBPs and NBPs by well diffusion assay, minimum inhibitory concentration (MIC), and minimum bactericidal concentration (MBC) determination. The effect of SNBPs on exposed bacteria was observed by scanning electron microscopy.

**Results:**

The UV-Vis of purified NBPs exhibited two absorption bands located at 550 nm and 849 nm with yield of bipyramidal particles more than 90%. The average size of NBPs was 76.33 ± 10.11 nm in length and 26.57 ± 2.25 nm in diameter, respectively, while SNBPs were prolongated in length and achieved 182.37 ± 21.74 nm with multi-branches protruding whole surface areas. In antibacterial evaluations, SNBPs and NBPs showed antibacterial activity with MIC of 6.25 μl/ml and 12.5 μl/ml, respectively, for MSSA while 12.5 μl/ml and 25 μl/ml, respectively, for MRSA. Besides, MBC values of SNBPs and NBPs were found to be 12.5 μl/ml and 25 μl/ml, respectively, against MSSA while 25 μl/ml and 50 μl/ml, respectively, against MRSA. Furthermore, scanning electron microscopy observation showed the mechanism that SNBPs damaged the outer membrane, released cytoplasm, and altered the normal morphology of MRSA and MSSA, leading to bacterial death.

**Conclusions:**

This report suggests that these SNBPs are potential antibacterial agents that can be applied as antibacterial materials to inhibit the growth of human bacterial pathogen infections related to antibiotic-resistant bacteria.

## Background

Antibiotic-resistant bacteria are becoming a global risk in recent years, and posing worldwide health faces challenges and threats. Antibiotic and microbial multidrug resistance is one of three top threats of global public health in the modern age beside climate change and noncommunicable diseases, according to the World Health Organization (WHO) [1]. Therein, the ESKAPE group (*Enterococcus*, *Staphylococcus*, *Klebsiella*, *Actinobacter*, *Pseudomonas*, *Enterobacter*) is the most concern [2]. Among them, *Staphylococcus aureus*, gram-positive bacteria, are common bacteria causing a variety of infectious diseases which spread in health care clinic and community [3]. Methicillin-resistant *Staphylococcus aureus* (MRSA), which was first described in 1960, is one of the most popular multidrug-resistant bacterial pathogens worldwide [4]. According to the Centers for Disease Control (CDC) report, MRSA is resistant to most of beta-lactam antibiotics including methicillin and other common antibiotics such as oxacillin, penicillin, and amoxicillin [5, 6]. There are two strains of MRSA infections: hospital acquired (HA) and community acquired (CA) [7, 8]. According to a research which was published in The Lancet in 2022, MRSA was the deadliest pathogen-drug combination globally [9]. The number of all-age MRSA deaths is largest in the Southeast Asia, East Asia, and Oceania super-region and is smallest in the Central Europe, Eastern Europe, and Central Asia super-region. In the community, MRSA most often causes skin infections; however, it causes pneumonia (lung infection) and other infections in some cases. In medical clinics, MRSA can lead to bloodstream infections caused by surgical equipment infections.

Current therapies in MRSA include newer antibiotics, antimicrobial photodynamic therapy, phage therapy, and nanomaterials. Vancomycin or daptomycin is first-line agents for treating MRSA [10, 11]. In addition, several synergistic antibiotics such as ceftaroline, linezolid, quinupristin-dalfopristin, telavancin, trimethoprim-sulfamethoxazole, and fosfomycin have been widely studied to treat MRSA infections. However, antibiotic resistance is developing due to over- prescription, overuse or shorter course of antibiotics. These lead to bacteria themselves alternate to limit the uptake of antibiotics, eliminate or destroy antibiotics. Other MRSA treatment is antimicrobial photodynamic therapy [12]. This method based on radical species and hydrogen peroxides, which produced by oxygen molecules under exposed energy, resulting in killed bacteria. Nevertheless, this method consumes expensive cost for MRSA treatment. Another method in MRSA treatment is bacteriophage therapy or phage therapy [13]. Phages were found that they are effective activity against MRSA samples. However, some studies indicated that this therapy may rise the resistant phenomenon after long usage. Therefore, another effective and cheap method for MRSA treatment is necessary. Nanotechnology, especially nanomaterials, is a promising therapeutic strategy and more widely applied because of its high efficacy and extraordinary therapeutic mechanism against microorganism [14, 15]. Nanoparticles such as gold nanoparticles, silver nanoparticles, and other oxide metal nanoparticles have the ability to defend against antibiotic-resistant mechanism of bacteria through some pathways: interacting and damaging membrane of bacteria, penetrating inside the cells, inactivating protein and enzyme, releasing free metal ions, or disrupting DNA synthesis [16, 17].

In recent reports, antibacterial activity of gold nanoparticles relates to a cell membrane disruption or decline in metabolism and transcription process of bacteria [18]. Herein, gold nanoparticles attach onto bacterial membrane and penetrate cytoplasm, resulting in inhibition of ATPase production as well as inhibiting the subunit of ribosome for tRNA binding, which lead to the breakdown in metabolism and transcription process [19]. According to these pathways, gold nanoparticles, which have large surface area, exhibit a higher probability of interaction with bacterial membranes [20]. Gold nanobipyramids (NBPs), which are penta-twinned crystalline structure with ten {111} facets, provide greatly antibacterial effect [21, 22]. Additionally, gold nanostars, which structure includes many spikes protruding around spherical core, show high antibacterial activity due to plenty of {111} facets [23]. Based on reports above, the combination of spikes and anisotropic gold nanoparticles could prepare a new family of spiked gold nanoparticles which engage to achieve higher antimicrobial effect. The first concept of growing tip on anisotropic gold nanoparticles was reported the formation of spiked gold nanorods [24]. Another spiked particles were spiked gold nanotriangles resulting from the growth of tip ultra-flat [25]. Both spiked gold nanoparticles were synthesized and applied in enhancing surface-enhanced Raman spectroscopy.

In this report, we introduce a preparation of spiked gold nanobipyramids (SNBPs). The high-density {111} facets of gold nanoparticles are synthesized based on traditional seed-mediated methods [26, 27]. Produced NBPs are purified to remove mostly spherical particles. The tips are grown directly on surfaces of nanobipyramidal particles in the presence of ascorbic acid and directing agents such as silver nitrate and cetyltrimethylammonium bromide. In addition, the report has performed antibacterial efficacy of SNBPs and NBPs against both MRSA and MSSA such as ﻿minimum inhibitory concentration (MIC) and minimum bactericidal concentration (MBC). Further, the effects of SNBPs on MRSA and MSSA were investigated by SEM observations.

## Methods

### Materials

Chloroauric (III) acid (HAuCl_4_xH_2_O, 52% Au basis), hexadecyltrimethylammonium bromide (CTAB, 98%), 3-(N, N-dimethyltetradecylammonio)propanesulfonate (SB3-14, 98%), ascorbic acid (C_6_H_8_O_6_, 99%), sulfuric acid (H_2_SO_4_, 99%), and sodium borohydride (NaBH_4_, 98%) were purchased from Sigma-Aldrich. Silver nitrate (AgNO_3_, 99%), hydrogen peroxide (H_2_O_2_, 30%), ammonia solution (NH_4_OH, 25%), cetyltrimethylammonium chloride solution (CTAC 25%), and polyethylene glycol (PEG 4000) were obtained from Merck. Deionized water (18 MΩ) was used throughout experiments. All chemical materials were GR grade.

The bacterial culture media were tryptic soy broth/agar (TSB/TSA). Ampicillin antibiotic and resazurin were obtained from Sigma, Merck. The test organisms used in this study were methicillin-resistant *Staphylococcus aureus* ATCC 43300 (MRSA) and methicillin-susceptible *Staphylococcus aureus* ATCC 33591 (MSSA).

### Spiked gold nanobipyramids preparation

**Fig. 1:**
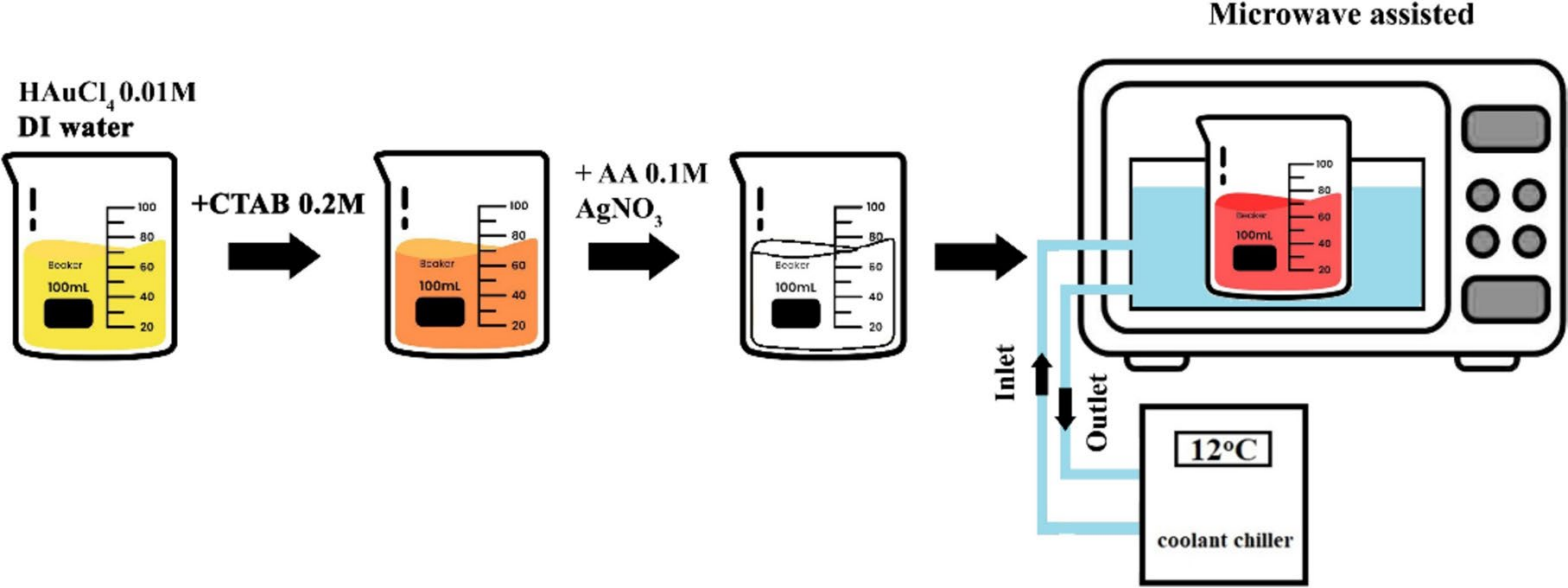
Schematic of NBPs preparation

The preparation of NBPs was carried out combining seed-mediated and microwave-assisted method. Firstly, 2 ml of HAuCl_4_ 0.25 × 10^−3^ M solution was reduced by 400 μl of cold NaBH_4_ 0.1-M solution in the presence of CTAB 0.1 M to make seed solution. This mixture was kept constantly at room temperature before using for the following step. For growth solution preparation, 500 μl of HAuCl_4_ 0.01 M was added into 9.5 ml of CTAB 0.1-M solution. Adjust pH to 3 using H_2_SO_4_ 20% solution, kept on adding 75 μl of AgNO_3_ 0.01 M and 75 μl of ascorbic acid (AA) 0.1 M, and color of mixture changed from brownish yellow to colorless. Finally, 35 μl of seed solution was dissolved rapidly into this mixture. The process forming gold nanobipyramidal particles is reacted under microwave assisted at room temperature (Fig. 1). The color of mixture turned from colorless to burgundy indicating that NBPs solution was obtained.

**Fig. 2:**
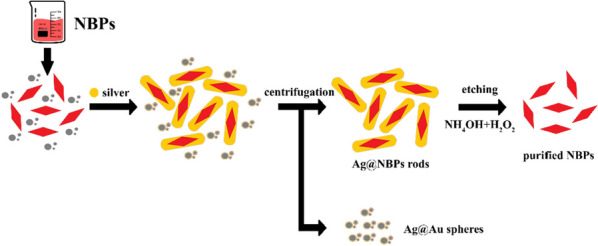
Schematic of NBPs purification

The purification of NBPs was displayed in Fig. 2. The core@shell of silver/NBPs nanorods (Ag@NBPs nanorods) were separated using centrifugation and redispersed in CTAB. The Ag coated outside NBPS were etched away using a mixture solution of NH_4_OH and H_2_O_2_ solution, resulting in pure nanobipyramidal particles. The purified NBPs were dispersed in CTAB 0.05 M for stable maintenance [28].

The synthesis of SNBPs was achieved according to the described protocol. Firstly, 500 μl of HAuCl_4_ 0.01-M solution was diluted in 4.25-ml deionized water (DI). Next, 3-ml CTAB 0.2 M and 2-ml SB3-14 0.2 M were added into the solution above. Then, 1000 μl of purified NBPs solution was injected into the mixture. Finally, various volumes of AgNO_3_ 0.01 M and 250-μl AA 0.1-M solution were added into mixture to investigate the effect of reduction agent on spiked formations onto surfaces of NBPs. The color of mixture turned to cobalt indicating the formed SNBPs.

### Characterization NBPs and SNBPs

NBPs and SNBPs were characterized by Jasco V-730 spectrophotometer in the wavelength range between 400 and 900 nm and scanning rate 200 nm/min. Crystal structure of NBPs and SNBPs was determined employing X-ray diffraction (XRD). The scanning was carried out in the 2-theta range of 20–80° using X-ray diffractometer Bruker D5005. Hitachi S-4800 was used to obtain scanning electron microscope (SEM) micrographs at an accelerating voltage of 10 kV. Transmission electron microscope (TEM) analysis was examined by JEM1010-JEOL. The average sizes of NBPs and SNBPs were calculated by ImageJ software (NHI Image), based on particles of each three samples from the TEM micrographs. All gold nano colloidal solutions were sonicated before measuring and examining.

### Screening of antibacterial properties

The antibacterial activity of SNBPs and NBPs was determined using an agar well diffusion method [29] against strains, namely, MRSA and MSSA. Tryptic soy agar (TSA) was used to streak the bacterial culture, followed by 24-h incubation at 37 °C. The actively growing cultures approximated cell density at 10^7^ CFU/ml by adjusting the spectrophotometer OD_600_ values to 0.1. The prepared culture suspensions of each tested strain (100 μl) were spread to the surface of TSA agar, followed by perforations which were made and loaded with 100 μl of tested SNBPs or NBPs (twofold serial dilutions concentration). Ampicillin was used as positive control, whereas distilled water was negative control. The plates were incubated at 37 °C for 24 h. The diameter of the zone of inhibitions was measured in mm. For each test, three replicates were performed. Each experiment was carried out three times as well as all collected data were calculated in statistical analysis as mean ± SD. All data were analyzed by ANOVA and Tukey’s test using Minitab 21.4, OriginPro 2022 software. The diameter zone of inhibition result was analyzed by ANOVA using mean and standard deviations. Significant differences were established for a probability level of 5% (*p* < 0.05).

### MIC and MBC determination of SNBPs and NBPs against MRSA and MSSA

The antibacterial effectiveness of SNBPs and NBPs against MRSA and MSSA were analyzed through determination of the MIC and MBC and through application of the broth microdilution assay using 96-well microtiter plates [30].

Briefly, after overnight growth of MRSA and MSSA at 37 °C, cell density was adjusted to OD_600_ = 0.1 (corresponding to ~10^7^ CFU/ml). The SNBPs or NBPs solutions were diluted using twofold serial dilution from 400 to 0.05 μg/ml in the same medium. Then 100 μl of SNBPs or NBPs solution and 100 μl of bacterial inoculum (1 × 10^7^CFU/ml) were added in the 96-well micro-titer plates. Media (broth) was used as positive control, while bacterial inoculum was negative control. After plate incubation at 37 °C for 24 h, resazurin (Sigma-Aldrich) was supplemented to all well and further incubated for 1–2 h for the observation of color change. On completion of the incubation, columns without color change (blue resazurin color remained unchanged) were scored as above the MIC value [31]. The lowest concentration of SNBPs or NBPs that inhibited bacterial growth was considered the MIC. After the MIC determination, 100 μl of aliquots from each well, which did not show any bacterial growth after incubation, was streaked onto TSA agar plates followed by incubation at 37 °C for 24 h. The lowest concentration which destroys 100% of the initial bacterial population showing no colonies on the TSA agar was recorded as the MBC [32].

### Tolerance level

The bacteria’s level of tolerance reveals whether SNBPs or NBPs are bacteriostatic or bactericidal. Herein, the ratio of MBC to MIC was used to calculate the MSSA and MRSA tolerance level in gold nanoparticles [33]. The above ratio greater than 16 considers gold nanoparticles as bacteriostatic, while the same less than 4 considers gold nanoparticles to possess bactericidal activity [34].

### Antibacterial test SEM observation

**Fig. 3:**
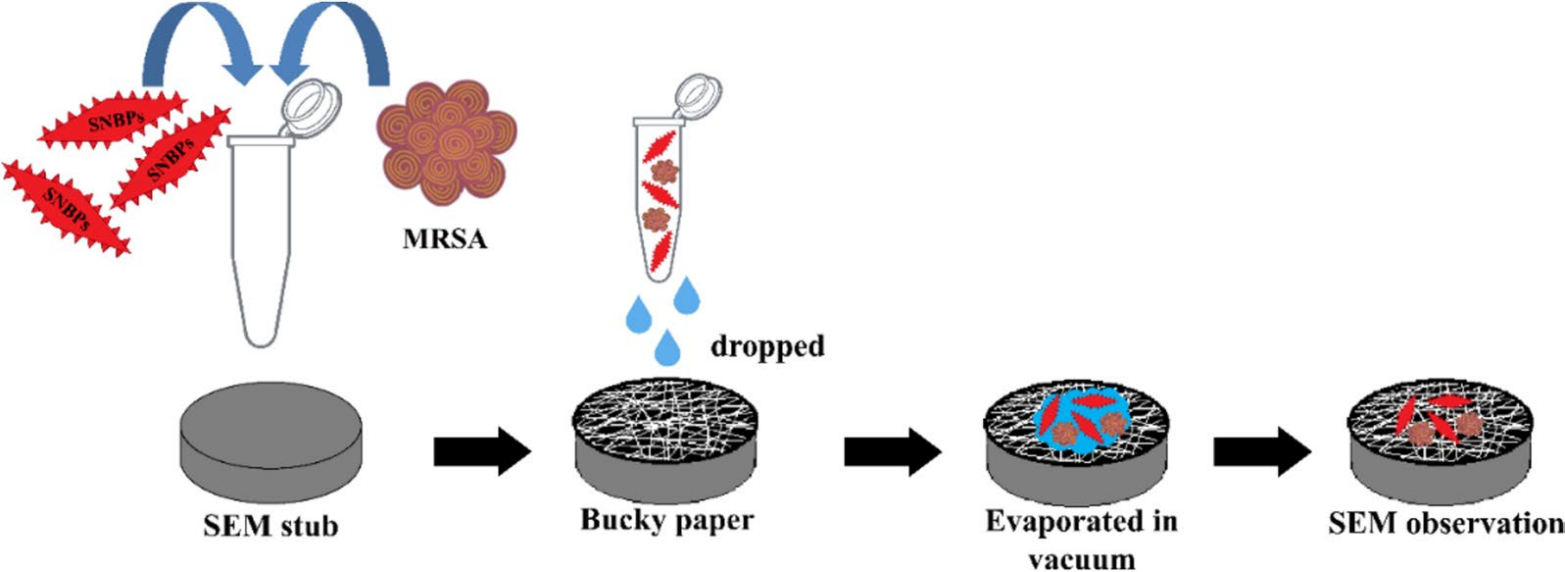
The procedure of sample preparation for observing bacteria specimen

Besides, a simple method was carried out to observe the mechanism of SNBPs killing bacteria using scanning electron microscope. The protocol to prepare the observing specimen was exhibited in Fig. 3. After MRSA and MSSA cultures were exposed with SNBPs in Eppendorf tubes, these mixtures were directly dropped on stubs and evaporated in vacuum at 40 °C without any fixative and dehydrating process. The SEM observation was carried out on Hitachi S-4800 equipment at accelerating voltage 3 kV.

## Results

### Characterization of NBPs

**Fig. 4:**
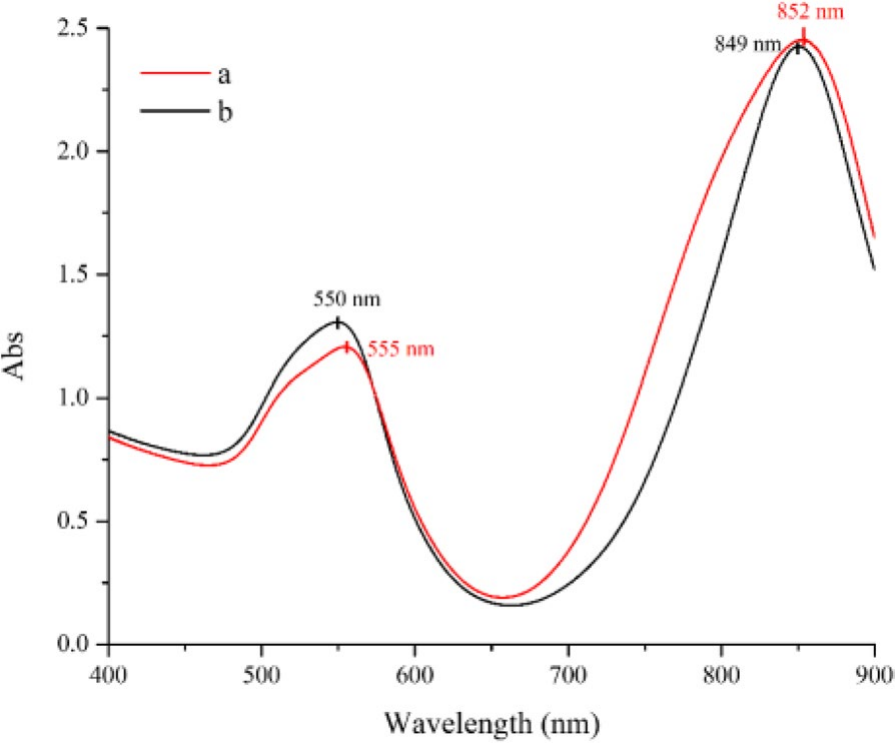
UV-Vis spectrum of produced (**a**) and purified NBPs (**b**)

**Fig. 5:**
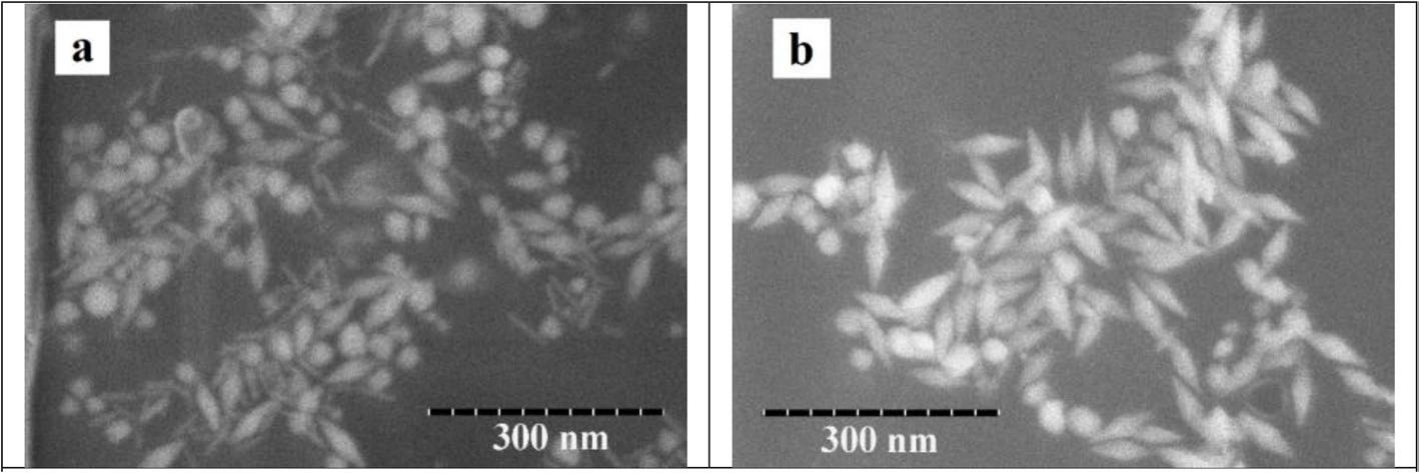
SEM micrographs of produced (**a**) and purified NBPs (**b**)

The UV-Vis spectrum of produced NBPs and purified NBPs was shown in Fig. 4. The produced NBPs had two absorption bands at 555 nm and 852 nm, while absorbance bands of purified NBPs were located at 550 nm and 849 nm. Besides, the full width at half maximum (FWHM) of purified NBPs was narrower than produced NBPs, indicating that contaminating particles such as spheres or rods were removed. This assessment was demonstrated by SEM micrographs in Fig. 5. The produced NBPs consisted of many spherical and nanorods particles. The purity of NBPs was calculated based on particles of each three sample from TEM micrographs which was by ratio of bipyramidal nanoparticles to total nanoparticles in each TEM micrograph. It was noticeable that the yield of purified NBPs was approximately 90%, and most of spheres and rods were removed.

### Characterization of SNBPs

**Fig. 6:**
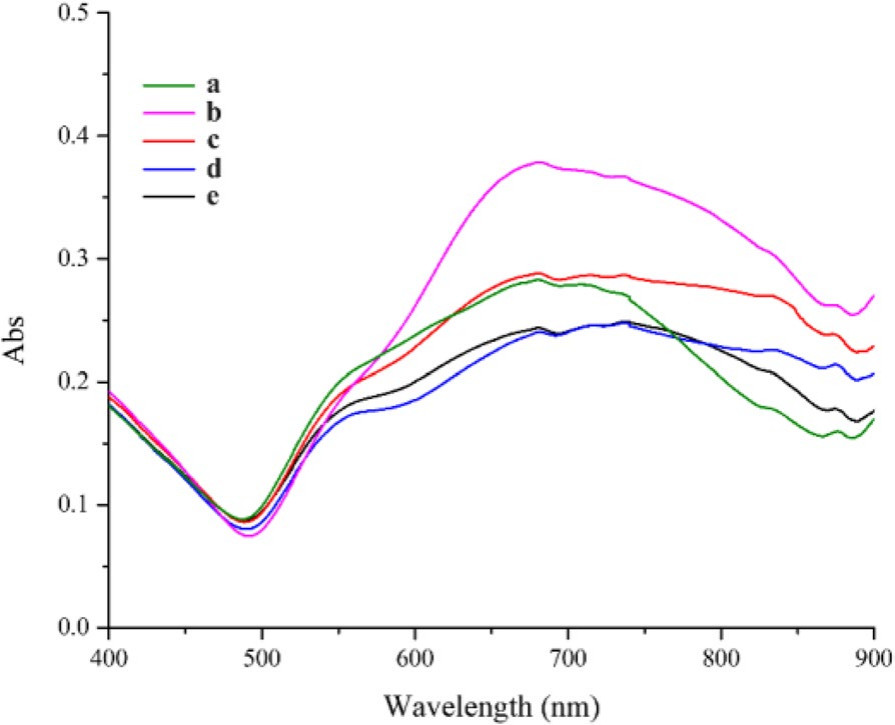
The UV-Vis results showed the effect of 0.01-M AgNO_3_ volumes on SNBPs formation. 0 μl (**a**). 25 μl (**b**). 50 μl (**c**). 75 μl (**d**). 100 μl (**e**)

**Fig. 7:**
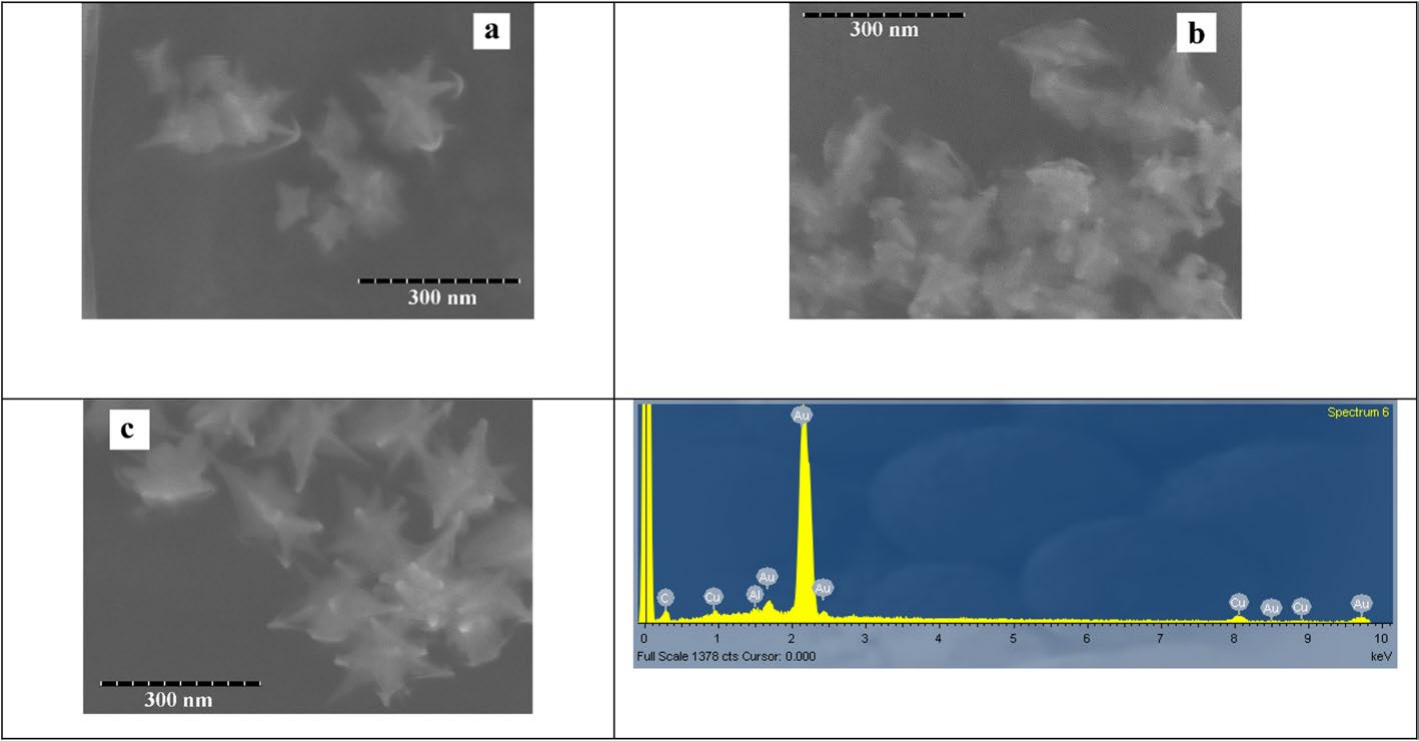
SEM micrographs of prepared SNBPs in different volumes of AgNO_3_. 0 μl (**a**), 25 μl (**b**), 100 μl (c), and EDX spectrum of produced SNBPs at 25 μl of 0.01-M AgNO_3_ (**d**)

Figure 6 showed the UV-Vis results of SNBPs synthesized in various 0.01-M AgNO_3_ volumes. In the absence of AgNO_3_, the absorption spectra were illustrated including a weak peak at 550 nm and a wide absorption ranging from 600 to 820 nm. In the presence of 25-μl AgNO_3_ 0.01 M, the absorption peak is located at 681 nm, while intensity reached the highest value. It was noticeable that UV-Vis results exhibited two peaks enclosing one peak at 554 nm and one broad absorbance region from 580 to 860 nm, while the intensity of absorption peaks decreased when kept on increasing volumes to 100 μl of 0.01-M AgNO_3_ solution. The first peak may be related to core, and the second one could be assigned to multi-branches of particles as SEM micrographs in Fig. 7. It was clear that only multibranched gold nanoparticles were formed without spiked bipyramidal particles in the AgNO_3_ absence (Fig. 7a). However, most bipyramidal particles were protruded spikes homogeneously whole surface area at 25 μl of 0.01-M AgNO3 solution (Fig. 7b). Nevertheless, morphology of NBPs trended towards multibranched gold nanoparticles as volumes of the AgNO_3_ were increased to 100 μl (Fig. 7c). The EDX spectrum in Fig. 7d revealed strong peak at 2 keV which indicates the presence of gold element.

### XRD analysis

**Fig. 8:**
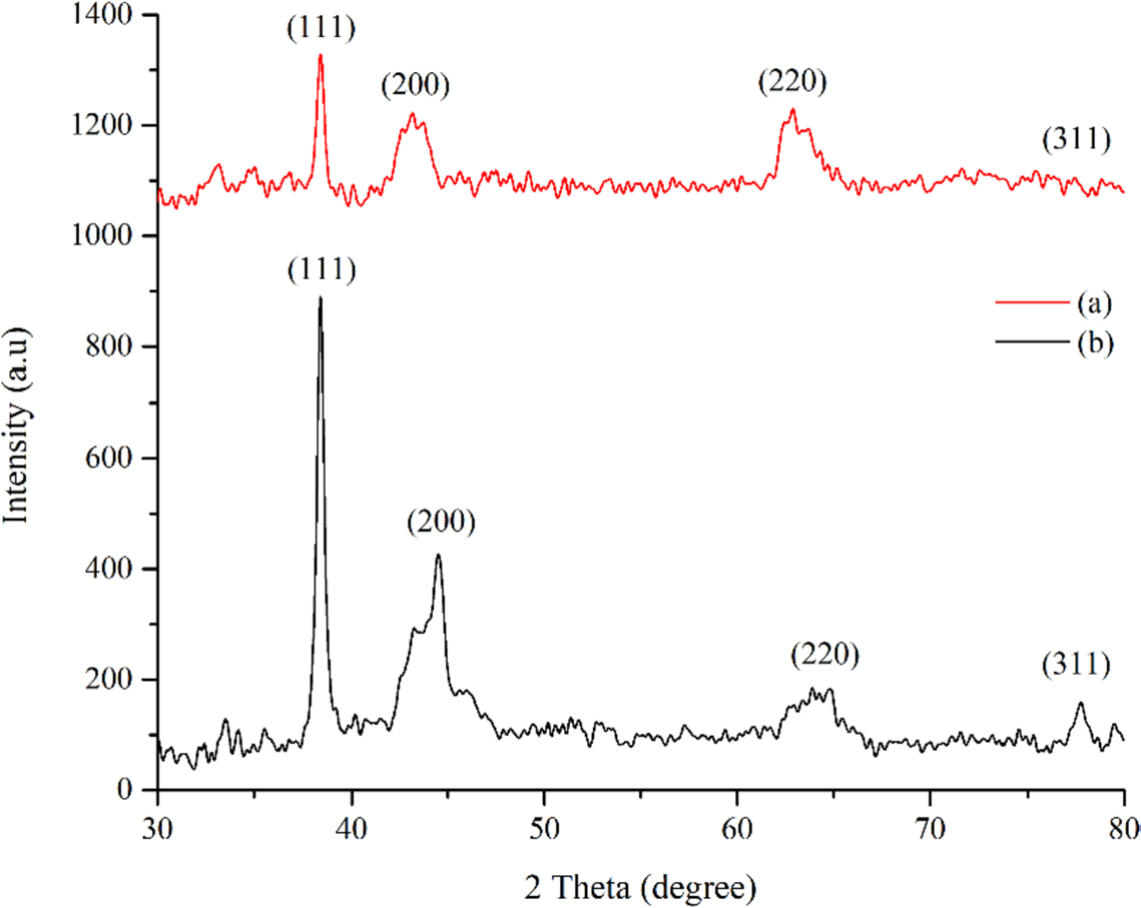
XRD patterns of NBPs (**a**) and SNBPs (**b**)

The XRD patterns of NBPs and SNBPs were shown in Fig. 8. Both recorded patterns exhibited peaks located at 38.4°, 44.5°, 64.8°, and 77.8°, which correspond to the exhibits of four diffraction peaks and corresponded to (111), (200), (220), and (311) planes of gold with face-centered-cubic (fcc) structural crystal, respectively (ICDD PDF card number 00-004-0784) [35]. It was clear that there was an intense peak located at 38.4° which was indexed to (111) plane. Besides, a moderate peak for (220) plane was observed at 44.5°. Additionally, there were another which appeared at 65.8° for (220) plane and a weakest peak at 77.8° corresponding to (311) plane. It was noticeable that the peak corresponding to (111) plane of SNBPs was about three times as intense as NBPs. It was indicated that the {111} facets density of SNBPs was more than NBPs [36, 37].

### Screening of antibacterial properties

The diameter of inhibition zones revealed that there is antibacterial potential of SNBPs and NBPs against both MSSA and MRSA (Table 1). In case of antibacterial activity, the developed SNBPs and NBPs exhibited little antibacterial effectiveness as compared to ampicillin. The outcomes revealed SNBPs and NBPs indicated growth inhibition in MRSA and MSSA, and the diameter of zone inhibition varied between 8.67 ± 0.58 nm and 15.67 ± 0.58 mm. Besides, it was found that inhibitory effect of SNBPs was slightly better than NBPs.

**Table 1:** Inhibiting zones of SNBPs and NBPs

Tested strains	Types gold nanoparticles	400 (μg/ml)	200 (μg/ml)	100 (μg/ml)	50 (μg/ml)	25 (μg/ml)	12.5 (μg/ml)
MSSA	**NBPs**	14.33^bcd^ ± 0.58	13^bcde^ ± 0.0	12^cde^ ± 1.00	11^cde^ ± 0.0	10.33^de^ ± 0.58	0^h^ ± 0.0
	**SNBPs**	15.67^abc^ ± 0.58	14.67^abc^ ± 0.58	14^bcd^ ± 0.0	13.33^bcde^ ± 0.58	12.33^cde^ ± 0.58	10.67^de^ ± 0.58
MRSA	**NBPs**	13.67^bc^ ± 1.52	12.67^bcde^ ± 0.58	11.67 ± 0.58	11^cde^ ± 0.58	10.33^de^ ± 0.58	8.67^f^ ± 0.58
	**SNBPs**	14.67^abc^ ± 0.58	13.33^bcde^ ± 0.58	12.67^bcde^ ± 0.58	12^cde^ ± 0.58	11.33^de^ ± 0.58	9.67^e^ ± 0.58

Superscript with different letters in the same column shows significant difference (*p* < 0.05). *SD* standard deviation

### MIC and MBC determination against MRSA and MSSA

**Fig. 9:**
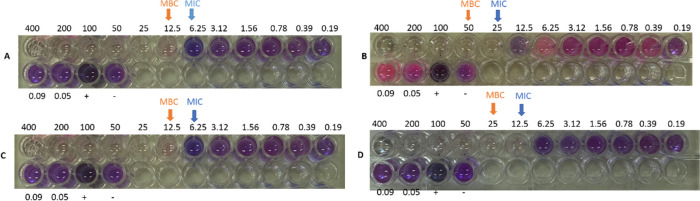
Microtiter plates showing the MIC and MBC of **A** SNBPs, **B** NBPs against MRSA, **C** SNBPs, **D** NBPs against MSSA

The MIC/MBC tests of SNBPs and NBPs which ranged from 0.05 to 400 μg/ml were shown in Fig. 9. The MIC values of SNBPs were 6.25 μg/ml, while the MBC values were found to be 12.5 μg/ml against both MSSA and MRSA. The MIC concentration of NBPs was detected as 12.5 and 25 μg/ml respectively, whereas the MBC values were found to be 25 μg/ml and 50 μg/ml, respectively, against both MSSA and MRSA strains (Table 2). Therefore, efficient bacterial killing of SNBPs was slightly higher than NBPs. According to Sayani Mitra et al. [33], it showed that Pal-α-MSH (11–13) was conjugated with gold nanoparticles of MIC, and the MBC value was 18 μM against both MSSA and MRSA. Therefore, our results were in line with the previous reports of antibacterial effectiveness.

**Table 2:** Minimal inhibitory concentration (MIC) and minimal bactericidal concentration (MBC) values against MSSA (ATCC 29213) and MRSA (ATCC 33591)

Types gold nanoparticles	MSSA			MRSA		
	MIC (μg/ml)	MBC (μg/ml)	Tolerance level	MIC (μg/ml)	MBC (μg/ml)	Tolerance level
SNBPs	6.25 ± 0.0	12.5 ± 0.0	2	6.25 ± 0.0	12.5 ± 0.0	2
NBPs	12.5 ± 0.0	25 ± 0.0	2	25 ± 0.0	50 ± 0.0	2

### Tolerance level

Because the ratios of MBC to MIC were double times on both strains, so the tolerance level of NBPs as well as SNBPs were twice. This data indicated the bactericidal properties of SNBPs and NBPs.

## Discussion

The effect of AgNO3 on spiked formation could be explained according to above reports [38, 39]. Ag^+^ ions are obligated to form spikes onto surface of gold nanorods and nanotriangles. It could be explained based on the electrostatic interaction between the negatively charged surfactant bilayers of the NBPs and the positively charged Au^3+^ and Ag^+^ ions. In the absence of AgNO_3_, bipyramidal gold nanoparticle roles as seeds and Au^+^ ions were reduced directly by AA, forming multibranched nanoparticle which was larger than bipyramidal gold nanoparticles as in SEM micrographs. In contrast, in the presence of AgNO_3_, Au^3+^ and Ag^+^ ions were combined with cationic SB3-14 bilayer over whole surface of NBPs through electrostatic interaction. Therein, Ag^+^ ions attached on certain facets and Au^3+^ ions reduced to form {111} facets protrusions in analogy to growth of gold nanorods or gold nanobipyramids [40, 41].

**Fig. 10:**
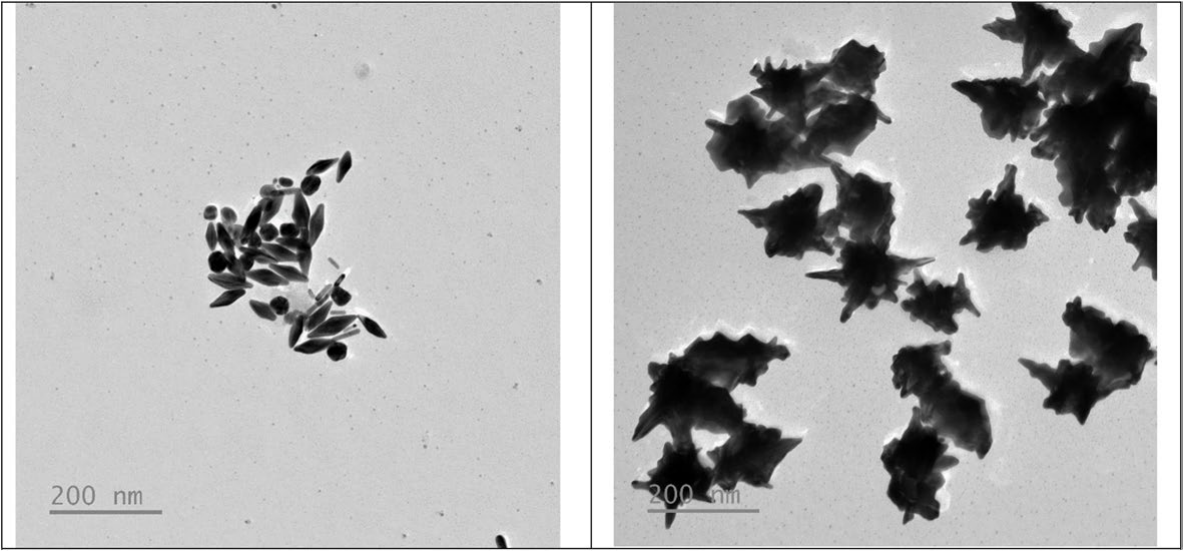
TEM micrographs of NBPs (**a**) and SNBPs (**b**)

TEM micrographs of NBPs and SNPS were carried out to calculate the average sizes using ImageJ software [42]. The results exhibited that NBPs were 76.33 ± 10.11 nm in length and 26.57 ± 2.25 nm in diameter, respectively, while SNBPs were 182.37 ± 21.74 nm in length with multi-branches protruding whole surface areas (Fig. 10).

**Fig. 11:**
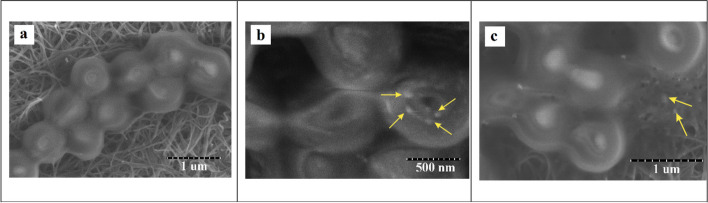
SEM micrographs showing the procedure of SNBPs killed MRSA. Normal MRSA’s cells (**a**). SNBPs penetrated inside bacterial cells (**b**). Bacteria were killed because of cytoplasm leakage (**c**)

**Fig. 12:**
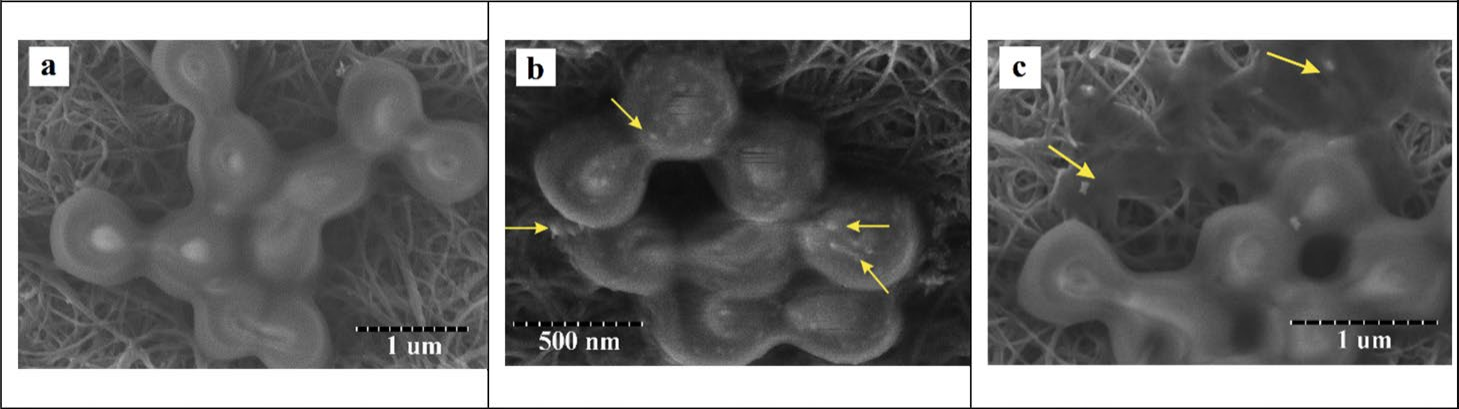
SEM micrographs showing the procedure of SNBPs killed MSSA. Normal MSSA’s cells (**a**). SNBPs penetrated inside bacterial cells (**b**). Bacteria were killed because of cytoplasm leakage (**c**)

The antibacterial activity of SNBPs was greater than NBPs because of their higher {111} facet density as performed by XRD results above. These were demonstrated in MIC/MBC results. Furthermore, the SEM micrographs on Figs. 11 and 12 exhibited the strategy SNBPs killing bacteria. The post growing MRSA and MSSA have stable and clear membranes indicated in Figs. 11a and 12a. After exposing MRSA and MSSA, spiked-bipyramidal gold particles attached onto bacterial cell membranes, and they penetrated inside bacterial cells (Figs. 11b and 12b). Finally, bacteria were killed because of cytoplasm leakage (Figs. 11c and 12c). According to results of antibacterial evaluation above, both antibacterial activities against MRSA and MSSA of SNBPs were more effective than NBPs. These results could be explained by interaction with bacterial membranes. As in many previous studies, metallic nanoparticles have some main mechanisms to kill bacteria such as the following: reactive oxidative species (ROS), releasing ion and interacting with the cell membrane resulted damaging membrane of bacteria and penetrating inside the cells, inactivating protein and enzyme, or disrupting DNA synthesis [43]. Beside ROS effect, the interaction and penetration of AuNPs with the cell membrane are supposed the pathway to kill microorganism. So, the antibacterial effect of AuNPs depends on attaching ability between AuNPs and cell membranes caused by facets which are determined by morphology of nanoparticles. Recent report shows that facets {111} could increase the affinity of the particles towards bacterial cell membranes [23] which boost the microorganism destroying potential. The bipyramidal gold nanoparticles mainly have ten {111} facets, while the spiked bipyramidal gold nanoparticles possess more {111} facets density than bipyramidal gold nanoparticles because of the combination of both. This can be demonstrated in XRD patterns in Fig. 8. Moreover, anisotropic gold nanoparticles which have many facets {111} may induce ROS generation leading to antibacterial effect increase [44]. The mechanism to damage bacterium could be briefed according to this pathway; after attaching onto bacterial membranes, spiked bipyramidal gold nanoparticles deform and break the cell membranes, leading to outside bacterial components leakage; they continuously penetrate the cytoplasm where they can inhibit protein process or generate ROS to poison bacteria, leading to bacterial death. Based on spectacular antimicrobial pathways, NBPs and SNBPs could resist the mechanisms, which bacteria can develop defense against antibiotics.

## Conclusions

This report presented a strategy for synthesis of SNBPs consisting preparation of NBPs using seed-mediated combined microwave-assisted method and spikes formation over whole surface area of bipyramidal nanoparticles process. NBPs and SNBPs were characterized using UV-Vis spectroscopy, SEM, TEM, and XRD.

Antibacterial evaluations exhibited that both NBPs and SNBPs showed impressive antibacterial activities against two strains of antibiotic-resistant bacteria MRSA and MSSA by antibacterial screenings and MIC/MBC methods. It was noticeable that SNBPs was more effective than NBPs in all investigations because of their high {111} facets density determined through XRD pattern. The procedure SNBPs, which killed bacterial cells, was established including attaching and deforming the cell membranes, penetrating the cytoplasm, and damaging bacteria because of outside bacterial components leakage by using SEM observation.

Therefore, it can be concluded SNBPs is successfully synthesized using a cheap and simple method. This nano material is a prospective agent for replacing antibiotics to solve antibacterial resistance in biomedical or other civil applications.

## Data Availability

10.6084/m9.figshare.23870814.v3

## Abbreviations

MRSA: Methicillin-resistant *Staphylococcus aureus*
MSSA: Methicillin-sensitive *Staphylococcus aureus*
NBPs: Gold nanobipyramids
SNBPs: Spiked gold nanobipyramids
MIC: Minimum inhibitory concentration
AA: Ascorbic acid
UV-Vis: Ultraviolet visible
XRD: X-ray diffraction
SEM: Scanning electron microscope
TEM: Transmission electron microscope

## References

[1] Mba IE, Nweze EI (2021). Nanoparticles as therapeutic options for treating multidrug-resistant bacteria: research progress, challenges, and prospects. World J Microbiol Biotechnol.

[2] Oliveira DMPD, Forde BM, Kidd TJ (2020). Antimicrobial resistance in ESKAPE pathogens. Clin Microbiol Rev.

[3] Guo Y, Song G, Sun M et al (2020) Prevalence and therapies of antibiotic-resistance in Staphylococcus aureus. Front Cell Infect Microbiol 10. 10.3389/fcimb.2020.00107 10.3389/fcimb.2020.00107 PMC7089872 32257966

[4] Larsen J, Raisen CL, Ba X (2022). Emergence of methicillin resistance predates the clinical use of antibiotics. Nature.

[5] Panchal VV, Griffiths C, Mosaei H (2020). Evolving MRSA: high-level β-lactam resistance in Staphylococcus aureus is associated with RNA polymerase alterations and fine tuning of gene expression. PLoS Pathog.

[6] Shang W, Rao Y, Zheng Y (2019). β-Lactam antibiotics enhance the pathogenicity of methicillin-resistant Staphylococcus aureus via SarA-controlled lipoprotein-like cluster expression. mBio.

[7] Tsouklidis N, Kumar R, Heindl SE (2020). Understanding the Fight against resistance: hospital-acquired methicillin-resistant Staphylococcus aureus vs. community-acquired methicillin-resistant Staphylococcus aureus. Cureus.

[8] Turner NA, Sharma-Kuinkel BK, Maskarinec SA (2019). Methicillin-resistant Staphylococcus aureus: an overview of basic and clinical research. Nat Rev Microbiol.

[9] Murray CJL, Ikuta KS, Sharara F (2022). Global burden of bacterial antimicrobial resistance in 2019: a systematic analysis. Lancet.

[10] Khan A, Wilson B, Gould IM (2018). Current and future treatment options for community-associated MRSA infection. Expert Opin Pharmacothe.

[11] Mahjabeen F, Saha U, Mostafa MN (2022). An update on treatment options for methicillin-resistant Staphylococcus aureus (MRSA) bacteremia: a systematic review. Cureus.

[12] Yeo WWY, Maran S, Kong AS-Y (2022). A metal-containing NP approach to treat methicillin-resistant Staphylococcus aureus (MRSA): prospects and challenges. Materials.

[13] Nandhini P, Kumar P, Mickymaray S (2022). Recent developments in methicillin-resistant Staphylococcus aureus (MRSA) treatment: a review. Antibiotics.

[14] Hussain S, Joo J, Kang J (2018). Antibiotic-loaded nanoparticles targeted to the site of infection enhance antibacterial efficacy. Nat Biomed Eng.

[15] Bekele T, Alamnie G (2022). Treatment of antibiotic-resistant bacteria by nanoparticles: current approaches and prospects. Ann Adv Chem.

[16] Balderrama-González A-S, Piñón-Castillo H-A, Ramírez-Valdespino C-A (2021). Antimicrobial resistance and inorganic nanoparticles. Int J Mol Sci.

[17] Okkeh M, Bloise N, Restivo E (2021). Gold nanoparticles: can they be the next magic bullet for multidrug-resistant bacteria?. Nanomaterials.

[18] Mobed A, Hasanzadeh M, Seidi F (2021). Anti-bacterial activity of gold nanocomposites as a new nanomaterial weapon to combat photogenic agents: recent advances and challenges. RSC Adv.

[19] Cui Y, Zhao Y, Tian Y (2012). The molecular mechanism of action of bactericidal gold nanoparticles on Escherichia coli. Biomaterials.

[20] Joshi AS, Singh P, Mijakovic I (2020). Interactions of gold and silver nanoparticles with bacterial biofilms: molecular interactions behind inhibition and resistance. Int J Mol Sci.

[21] Mehere A, Chaure NB (2020). Precisely controlled shape and size of gold nanostructures by seed-mediated reduction reaction method. Appl Phys A.

[22] Yougbaré S, Chou H-L, Yang C-H (2021). Facet-dependent gold nanocrystals for effective photothermal killing of bacteria. J Hazard Mater.

[23] Ray P, Lodha T, Biswas A (2022). Particle specific physical and chemical effects on antibacterial activities: a comparative study involving gold nanostars, nanorods and nanospheres. Colloids Surf A Physicochem Eng Asp.

[24] Aldeanueva-Potel P, Carbó-Argibay E, Pazos-Pérez N (2012). Spiked gold beads as substrates for single-particle SERS. ChemPhysChem.

[25] Liebig F, Sarhan RM, Bargheer M (2020). Spiked gold nanotriangles: formation, characterization and applications in surface-enhanced Raman spectroscopy and plasmon-enhanced catalysis. RSC Adv.

[26] Chateau D, Liotta A, Vadcard F (2015). From gold nanobipyramids to nanojavelins for a precise tuning of the plasmon resonance to the infrared wavelengths: experimental and theoretical aspects. Nanoscale.

[27] Li Q, Zhuo X, Li S (2015). Production of monodisperse gold nanobipyramids with number percentages approaching 100% and evaluation of their plasmonic properties. Adv Opt Mater.

[28] Qiao J, Li X, Qi L (2022). Fluorescent polymer-modified gold nanobipyramids for temperature sensing during photothermal therapy in living cells. Chin Chem Lett.

[29] Balouiri M, Sadiki M, Ibnsouda SK (2016). Methods for in vitro evaluating antimicrobial activity: a review. Chin J Pharm Anal.

[30] Methods for dilution antimicrobial susceptibility tests for bacteria that grow aerobically (2018) https://clsi.org/standards/products/microbiology/documents/m07/. Accessed 11 Jan 2018.

[31] Elshikh M, Ahmed S, Funston S (2016). Resazurin-based 96-well plate microdilution method for the determination of minimum inhibitory concentration of biosurfactants. Biotechnol Lett.

[32] Mondal AH, Yadav D, Mitra S, Mukhopadhyay K (2020). Biosynthesis of silver nanoparticles using culture supernatant of Shewanella sp. ARY1 and their antibacterial activity. Int J Nanomed.

[33] Mitra S, Mondal AH, Mukhopadhyay K (2022). Mitigating the toxicity of palmitoylated analogue of α-melanocyte stimulating hormone(11–13) by conjugation with gold nanoparticle: characterisation and antibacterial efficacy against methicillin sensitive and resistant Staphylococccus aureus. World J Microbiol Biotechnol.

[34] Oliveira MJ, de Almeida MP, Nunes D et al (2019) Design and simple assembly of gold nanostar bioconjugates for surface-enhanced raman spectroscopy immunoassays. Nanomaterials 9:1561. 10.3390/nano9111561 10.3390/nano9111561 PMC6915668 31689919

[35] Ogundare OD, Akinribide OJ, Adetunji AR (2019). Crystallite size determination of thermally deposited gold nanoparticles. Procedia Manuf.

[36] Kalyan Kamal SS, Vimala J, Sahoo PK (2014). A green chemical approach for synthesis of shape anisotropic gold nanoparticles. Int Nano Lett.

[37] Fang C, Zhao G, Xiao Y (2016). Facile growth of high-yield gold nanobipyramids induced by chloroplatinic acid for high refractive index sensing properties. Sci Rep.

[38] Koetz J (2020) The effect of surface modification of gold nanotriangles for surface-enhanced raman scattering performance. Nanomaterials 10:2187. 10.3390/nano10112187 10.3390/nano10112187 PMC7694140 33147806

[39] Liebig F, Sarhan RM, Schmitt CNZ (2020). Gold nanotriangles with crumble topping and their influence on catalysis and surface-enhanced Raman spectroscopy. ChemPlusChem.

[40] Orendorff CJ, Murphy CJ (2006). Quantitation of metal content in the silver-assisted growth of gold nanorods. J Phys Chem B.

[41] Li X, Yang Y, Zhou G (2013). The unusual effect of AgNO3 on the growth of Au nanostructures and their catalytic performance. Nanoscale.

[42] Razali NL, Morsin M, Nafisah S (2020). Formation of anisotropic gold nanoparticles on indium tin oxide substrates as a plasmonic sensing material. Nanomater Nanotechnol.

[43] Shamaila S, Zafar N, Sharif R (2018) Antibacterial activity of metallic nanoparticles. Bacterial pathogenesis and antibacterial control. InTech. 10.5772/intechopen.72526

[44] Piktel E, Suprewicz Ł, Depciuch J (2021). Varied-shaped gold nanoparticles with nanogram killing efficiency as potential antimicrobial surface coatings for the medical devices. Sci Rep.

